# Trapping White-Tailed Deer (Artiodactyla: Cervidae) in Suburbia for Study of Tick–Host Interaction

**DOI:** 10.1093/jisesa/ieaa044

**Published:** 2020-11-02

**Authors:** Patrick Roden-Reynolds, Erika T Machtinger, Andrew Y Li, Jennifer M Mullinax

**Affiliations:** 1 Department of Environmental Science and Technology, University of Maryland, College Park, MD; 2 Agriculture Research Service, Invasive Insect Biocontrol and Behavior Laboratory, Office of National Programs, United States Department of Agriculture, Beltsville, MD; 3 Department of Entomology, 4 Chemical Ecology Laboratory, Pennsylvania State University, University Park, PA

**Keywords:** *Odocoileus virginianus*, live capture, tick, vector surveillance, capture success

## Abstract

Live capture of white-tailed deer (*Odocoileus virginianus*) (Zimmermann, 1780) is often necessary for research, population control, disease monitoring, and parasite surveillance. We provide our deer trapping protocol used in a tick-host vector ecology research project and recommendations to improve efficiency of deer trapping programs using drop nets in suburban areas. We captured 125 deer across two trapping seasons. Generally, lower daily minimum temperatures were related to increased capture probability, along with the presence of snow. Our most successful trapping sites were less forested, contained more fragmentation, and greater proportion of human development (buildings, roads, recreational fields). To improve future suburban deer trapping success, trapping efforts should include areas dominated by recreational fields and should not emphasize remote, heavily forested, less fragmented parks. Concurrently, our study illustrated the heterogeneous nature of tick distributions, and we collected most ticks from one trapping site with moderate parameter values between the extremes of the most developed and least developed trapping sites. This emphasized the need to distribute trapping sites to not only increase your capture success but to also trap in areas across varying levels of urbanization and fragmentation to increase the probability of parasite collection.

Blacklegged ticks *Ixodes scapularis* Say 1821 (Ixodida: Ixodidae) are the vector of *Borrelia burgdorferi* Johnson 1984 emend. Baranton 1992 (Spirochaetalis: Spirochaetaceae), the causative agent of Lyme disease in North America. Additionally, Lone star ticks *Amblyomma americanum* Linnaeus 1758 (Ixodida: Ixodidae) are commonly reported in southeastern regions and are the vectors of several pathogens such as *Ehrlichia chaffeensis* (Rickettsiales: Anaplasmataceae), the causative agent of Ehrlichiosis (Masters et al. 2008). Vector-borne disease cases have tripled in just over a decade in the United States with Lyme disease accounting for most of these cases (Rosenberg et al. 2018). In urban and suburban areas, the presence of domestic pets, proximity to human recreational areas, and interspersion of natural habitats and developed habitats increase the risk of exposure to pathogens or zoonotic diseases (Hollis-Etter et al. 2019).

White-tailed deer, *Odocoileus virginianus* (Zimmermann, 1780), are a keystone host of the blacklegged tick, *Ixodes scapularis* (Barbour and Fish 1993). Surveillance of ticks on hosts is an important component of understanding the ecology of this species. Collecting biological samples for vector and disease monitoring for wildlife and human health is becoming more common and often requires live capture of the specific host species (Bloemer et al. 1988, Merrill et al. 2018, Hollis-Etter et al. 2019), but because capture of vertebrate hosts can be complicated; entomologists and disease ecologists have historically relied on ticks recovered from hunter harvests. However, recovery of ticks from these harvests introduces several variables, including time of death, access to the deer body which may be eliminated with an arrow or bullet, and is limited by hunting season regulations. In addition, parasite collections of hunter-harvested animals may not permit assessment of a specific area like a neighborhood or park, especially if those target areas are urban or suburban where hunting seldom occurs.

Live capture of deer for wildlife research and management is costly and time-demanding (Jones and Witham 1995, Mayer et al. 1995). Several studies have evaluated the cost and labor required for trapping efforts. Reported costs varied immensely from $21/animal up to $3,200/animal depending on capture success, initial start-up costs, and labor hours (Clark et al. 1981, Conner et al. 1987, Bryant et al. 1993, Jordan et al. 1993, Clark 1995, Jedrzejewski and Kamler 2004, Cosgrove et al. 2012). Although costly and laborious, collection of active ticks and other ectoparasites are best done on live animals (Rutberg et al. 2013, Tsunoda 2014, Merrill et al. 2018). Using primarily cost and time, previous studies have evaluated if trapping programs were feasible as a population control strategy, but few studies have evaluated what factors affect capture success. Given the cost and time investment, maximizing your capture success is crucial.

In past studies, habitat characteristics, immediate land use, deer density, and deer behavior were main contributors to overall trapping success (Garrott and White 1982, Webb et al. 2008, Hiller et al. 2010). Mayer et al. (1995) reported better success rates when capturing deer at high densities. Generally, single bait sources had limited effect on trapping success in comparison to the habitat quality or home range location (Kilpatrick and Stober 2002, Campbell et al. 2006, Barrett et al. 2008, Webb et al. 2008). Hiller et al. (2010) evaluated the effect of various environmental variables on capture success, reporting better capture probability in cold, snowy conditions in mid-western areas. However, in that study, deer restricted movements and foraging behavior when weather worsened. Barrett et al. (2008) reported local differences in capture success between sites, but no studies have formally evaluated habitat characteristics or differences in land use near trap sites that might affect capture success.

Direct comparisons between habitat characteristics from past studies proves difficult because the scale of study sites differ from 10 to 100,000 ha and lack information on exact trap locations. Urban landscapes and habitats are highly variable, fragmented, and change drastically in short distances. Completing a small-scale analysis of land use near trapping sites to identify trapping hot spots can greatly inform research efforts to capture deer.

In this article, we document trapping success in two trapping seasons in a highly suburban area to evaluate habitat characteristics, land use features (land cover, crop fields, buildings, roads, recreational fields), and assess the relationship between weather variables (temperature, daily precipitation, daily snowfall, daily snow depth) and deer capture success. Given the high cost of trapping deer, the goal is to provide trapping protocols, guidelines, and considerations to make urban trapping more efficient, especially in instances when vector surveillance or GPS deployment to better understand urban vector host ecology is the primary motivation for trapping.

## Methods

### Study Area

**Fig. 1.**
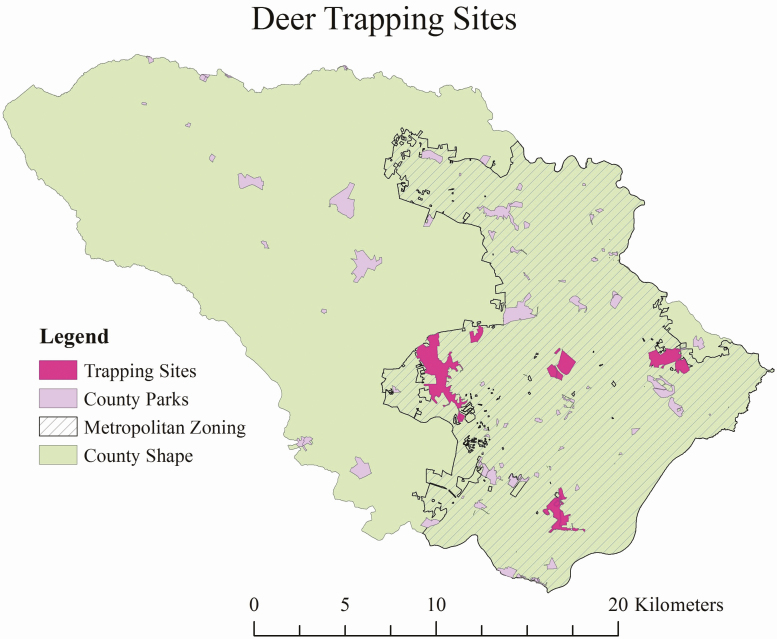
Map of Howard County MD and metropolitan zone containing the five selected county parks. Other county parks are depicted as purple polygons. The most southern park is Wincopin Trails/Savage Park. The four parks above Wincopin Trails from right to left are Middle Patuxent Environmental Area, Cedar Lane Park, Blandair Regional Park, and Rockburn Branch Park.

The current study is part of an ongoing United States Department of Agriculture-Agriculture Research Service (USDA-ARS) project to suppress tick populations. A primary objective of the Areawide Tick Control Project was to collect data from and deploy GPS tracking collars on deer at each park. The GPS collars would facilitate a better understanding of the vector host’s usage of highly suburban areas. White-tailed deer trapping was conducted in five county parks within the metropolitan zone of Howard County, Maryland (Fig. 1). The metropolitan area of Howard County is characterized by 221 residential properties/km^2^ with average lot sizes ranging from 0.05 to 3.3 ha, which falls within the parameters used to define suburban areas (Brown et al. 2005, Hansen et al. 2005).

The 2017 trapping season occurred February–April and the 2018 season occurred January-April. Parks where trapping occurred ranged in size from 37 to 418 ha and estimated deer densities within the parks widely ranged from 12.5 to 174 deer/km^2^ (Table 1). All deer density surveys were conducted with Forward Looking Infrared helicopter surveys by county Recreation and Parks personnel and county police. Cedar Lane Park, Middle Patuxent Environmental Area (MPEA, henceforth), and Wincopin Trails/Savage Park area were trapped during the 2017 season and part of the 2018 season. Rockburn Branch Park and Blandair Regional Park were added to the trapping schedule in the 2018 season. All Parks contained a mixture of developed amenities for recreational use, trails, and open space, defined as forest cover or undeveloped grassland cover. Cedar Lane Park is a 37 ha area characterized by paved trails, athletic fields, picnic areas, and limited open space. The MPEA is a 418 ha park with an unpaved trail system. This park is largely open space maintained as mature forest stands and patches of protected early successional habitat. The Wincopin Trails system (115 ha) is directly adjacent to Savage Park (28 ha) and were analyzed as one unit. Together this area is characterized by paved and unpaved trails, mostly mature forest open space, and athletic fields located in Savage Park. Rockburn Branch Park is a 168 ha semi-wooded park with 14.5 km of paved pathways and unpaved trails. This park has several recreational fields and playgrounds, including a Frisbee golf course. Blandair Regional Park is divided into two properties by a major highway, totaling 119 ha. The southern property (58 ha) is mainly recreational fields and amenities. We trapped deer on the northern property (60.7 ha), which contains unpaved trails and primarily late successional open space.

**Table 1.** Summary of the five county parks used as deer trapping sites in Howard County Maryland

Trapping site	Size (ha)	Amenities	Density deer/km^2^		Population management
			2017	2018	
Cedar Lane Park	37.6	Athletic fields, storage facility, picnic area, paved trails, playgrounds	N/A	N/A	None
Middle Patuxent Environmental Area	418	Unpaved trails	41	21	Managed hunting
Wincopin Trails/Savage Park^*a*^	143	Paved/unpaved trails, athletic fields	12.5^*b*^	N/A	Managed hunting and sharpshooting
Rockburn Branch Park	168	Disc golf course, athletic fields. storage facilities, playgrounds	17	61.9	Sharpshooting
Blandair Regional Park	60.7	Historic farm estate, unpaved trails	N/A	174	Managed hunting and sharpshooting

^*a*^Wincopin Trails and Savage Park are directly adjacent recreational areas.

^*b*^Deer density was only calculated for Savage Park in 2017 not Wincopin Trails.

The County Recreation and Parks Department implement deer population control at various parks via sharpshooting or managed hunts. Sharpshooting is conducted at night over bait piles by licensed marksmen. Managed hunts are restricted to shotgun and archery hunting by registered public participants. MPEA, Blandair (north), and Wincopin Trails area have had annual managed hunts since 1998, 2003, and 2014 respectively. Sharpshooting has occurred at Rockburn Branch park, Savage Park area, and Blandair Regional Park since 2007. No population control is conducted at Cedar Lane Park (Table 1). All park properties are bordered directly by suburban neighborhoods and commercial buildings.

### Trapping Methods

Deer were captured using drop nets (15.2 × 15.2 m) and box traps (0.9 m Width × 1.22 m Height × 1.83 m Length) (Wildlife Capture Services, Flagstaff, AZ) baited with whole kernel corn and apples. Exact drop net placement within the site was selected to reduce interference with human recreational activity while maintaining ease of vehicle access. An area large enough for the net was cleared of large debris and special care was taken to remove glass, metal litter, and rocks. After pre-baiting for 3 d, the net was erected and monitored with a Moultrie M-888 camera trap (Moultrie Feeders, Birmingham, AL) to determine group size and frequency of visits from deer. Once a net had deer visiting daily, a hunting blind was erected >25 m from the net. During each trapping event, technicians would wait in the hunting blind and drop the net via remote control once a deer was positioned under the net. In addition to drop netting, four box traps were placed in areas of high deer activity but also hidden from human view to reduce interference. After box traps were placed, the area inside and directly outside the entrance were baited. In addition to Moultrie camera traps, we used SPYPOINT Link-3G (GG Telecom, Indianola, IA) cellular cameras to monitor box trap activity allowing for immediate alerts when an animal was captured. Trap doors were tied open for approximately 2 wk until deer became familiar with the bait and entered the trap daily. We modified our box trap trigger wires to stand at least 30 cm above the ground to avoid false triggers from non-target animals. Box traps were set in the evening and checked once a day at dawn. Box traps were not permitted to be set for capture while the parks were open due to concerns of public interference even though camera trap data showed deer activity at box traps throughout the day.

When an animal was identified under a drop net, the field crew activated the net, physically restrained the animals, and anesthetized animals by hand syringe in the gluteal muscle mass using BAM (Wildlife Pharmaceuticals, Windsor, CO) (Miller et al. 2009, Siegal-Willott et al. 2009). The fixed-dose BAM formulation contained 27.3 mg of Butorphanol, 9.1 mg of Azaperone, and 10.9 mg of Medetomidine per 1 ml of solution. BAM fixed-dose volumes were administered based on sex and estimated weight according to manufacturer recommendations. After injection, face blinds were applied, and deer were moved onto a tarp for processing. We placed a DuFlex medium ear tag (Valley Vet Supply, Marysville, KS) on the right ear of each deer, providing contact info and a chemical warning. Animals were maintained in sternal recumbency with the head elevated above the rumen and nose oriented downward throughout processing. Time of injection was recorded as Time 0. Physiological data was collected at 5-min intervals for a 20-min period. This included respiration rate (in breaths per minute BPM) as determined by counting chest excursions, rectal temperature, and oxygen saturation (SpO_2_) using a SurgiVet v1030 portable pulse oximeter with a tongue sensor (Smiths Medical, Dublin, OH). During the processing period, we sexed each individual and estimated age by examining tooth wear and replacement (Severinghaus 1949). Deer were categorized based on age as fawns (≤ 1 yr old) or adult (> 1 yr old). Each deer was examined for ticks by brushing back the fur then visually and tactilely searching primarily around the ears, head, and anus (Luckhart et al. 1992). Ticks were opportunistically collected from the axilla and inguinal regions. Ticks were removed with forceps and placed into vials with 90% ethanol for later identification. Every effort was made to maintain deer body temperature within normal limits. In warmer weather (ambient temperature over 15°C), a ground tarp was not used, and isopropyl alcohol was applied to the ears, axilla, and genital area. Ice was also placed around the abdomen of the individual. If body temperature decreased in cold temperatures, deer body temperature was normalized with space blankets and in extreme instances, the rate of heat loss was slowed by having team member maintain physical bodily contact with the deer under the blanket. After a minimum 20-min processing period, BAM was reversed with intramuscular administration of Atipamezole (25 mg/ml) and Naltrexone (50 mg/ml) (Wildlife Pharmaceuticals, Windsor, CO) in amounts based on initial injection amounts of BAM. The reversal agent was given in the contralateral gluteal muscle mass from the BAM injection. Time to sedation and recovery were recorded. Deer were immediately released after recovery and monitored until they exited the area (Supp Material [online only]).

Trapping was canceled if temperatures dropped below −12°C to ensure safety of captured individuals that may be stressed from the cold or poor body conditions. Trapping was also canceled on extremely windy nights or during severe storms to ensure crew safety. The trapping protocol was approved by the United States Department of Agriculture Animal Care and Use Committee (IACUC approval #16-024).

### Capture Success

We calculated trapping effort by counting all trapping events each day for both trapping methods. A drop net trapping event occurred when crew members activated a drop net regardless of capturing deer. For box trapping, a trapping event occurred when crews set the traps in the evening and checked them the following morning. Trapping effort accounts for multiple teams at different parks or the same park for drop netting each night. Most nights, we used two separate trapping crews working in two locations for drop netting to increase chances of successful captures during the season.

A successful trapping event occurred when at least one deer was caught under the drop net or in a box trap for that trap event. Only one successful trapping event was recorded even if multiple deer were trapped at the same time. On a few occasions, very small deer would be captured in a box trap but would be released without processing. Only deer that were captured and processed (given immobilizing agents and an ear tag) were recorded as captures. We calculated capture success as the number of successful trapping events divided by the total trap effort for each park. We also calculated the number of deer captured per trap night as another measure of trapping success (Morgan and Dusek 1992, Naugle et al. 1995, Barrett et al. 2008, Hiller et al. 2010, Cosgrove et al. 2012).

### Spatial Analysis

**Fig. 2.**
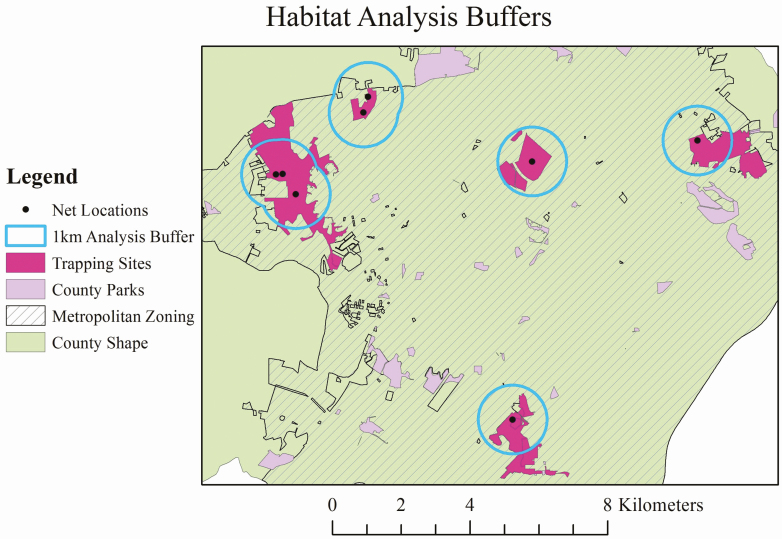
Map of Howard County Maryland with five selected parks and specific trapping sites that have 1,000 m buffer radius surrounding trapping sites. All GIS calculations were conducted within the buffers. Buffers that overlapped were merged as one.

We analyzed the habitat and land use characteristics immediately surrounding each trapping site. Box traps were excluded from habitat analysis because of very low capture rates. White-tailed deer in suburban areas exhibit high site fidelity and comparatively small home ranges (Swihart et al. 1995, Porter et al. 2004, Rhoads et al. 2010, Kilpatrick et al. 2011). Cornicelli et al. (1996) found that deer remained within 1 km of the trap locations. Therefore, we created a 1,000 m radius buffer around the center of drop net trapping sites (Fig. 2). For those parks with multiple trap sites that had overlapping buffers, buffers were merged.

We chose several variables to include in the habitat and land use analysis such as, land cover, distance to agriculture, amount of buildings (residential properties, park facilities, businesses), recreational fields, as well as density of roads and streams. We measured Euclidean distance for important features such as roads, buildings, recreational fields, and streams to compare between trapping buffers and specific net locations. We evaluated habitat by quantifying the area of land cover or croplands using land classifications schemes from National Land Cover Data (NLCD) 2016 and CropLand Data 2017–2018 (USDA NASS 2017, Yang et al. 2018). Four classification descriptions developed from the NLCD data set were grouped as follows: Urban cover (Developed Open Space; Developed Low/Medium/High Intensity; Barren Land), Forest Cover (Deciduous Forest, Evergreen Forest; Mixed Forest; Woody Wetlands), Shrub/Grassland Cover (Shrub/Scrub; Grassland/ Herbaceous; Pasture/Hay; Cultivated Crops; Emergent Herbaceous Wetlands) and Water. Forest edge density, patch density, and landscape division index was calculated using Fragstats software by extracting forest land cover class from NLCD dataset for analysis (McGarigal et al. 2012, Walter et al. 2018). All county-level feature data was sourced from Howard County GIS Data Download and Viewer.

### Statistical Analysis

Pearson’s χ ^2^ test was used to evaluate the relationship between capture success and trap site. Weather variables were gathered from a local weather station during the period of trapping (Baltimore Washington International Airport, Baltimore MD, NOAA). Habitat and weather variables were tested for multi-collinearity using Spearman’s correlation coefficients. Any pairs with *r* ≥ 0.7, required that one of the variables would be removed from the model. A stepwise general linear regression model using backward elimination with replacement was used to evaluate the relationship between daily capture success, weather covariates and spatial attributes of the trapping sites. The dependent variable was assumed to have a binomial distribution; thus, we used binomial family in the GLM model. Model selection was completed based on the AIC criterion. All analyses were performed using program R (R Core Team 2018).

## Results

### Trapping Results

We captured a total of 125 white-tailed deer (63 males, 62 females) during two trapping seasons using drop nets and box traps. In 2017, we captured 55 deer using drop nets and four deer using box traps. In 2018, we captured 63 deer using drop net and three using box traps. Overall, we captured 29 deer at Cedar Lane Park, 17 at Middle Patuxent Environmental Area, 29 deer at Wincopin Trails, 20 deer at Blandair Regional Park, and 30 deer at Rockburn Branch Park The only box trap captures occurred at Cedar Lane (*n* = 5) and MPEA (*n* = 2) (Table 2). Six of seven box trap captures were male. Only one of 125 captured deer were euthanized on site due to a broken back leg sustained during trapping. No mortality was attributed to the use of immobilizing drugs. GPS collaring was an objective of our study; thus, 50 of the 125 deer were monitored via radio telemetry for at least 30 d and no deaths were directly attributed to capture myopathy.

**Table 2.** White-tailed deer drop net captures at five county parks in Howard County, Maryland 2017–2018

Trapping sites	Year	Total captures	Trap events	Successful trap events	Overall capture success %	Deer/trap event
Cedar Lane	2017	26	24	10	46.2	0.92
	2018	3	2	2		
MPEA^*a*^	2017	12	26	9	28.2	0.38
	2018	5	13	2		
Wincopin Trails/Savage Park	2017	21	28	12	35.3	0.57
	2018	8	23	6		
Blandair Regional Park	2017	N/A	N/A	N/A	40	1
	2018	20	20	8		
Rockburn Branch Park	2017	N/A	N/A	N/A	40.6	0.94
	2018	30	32	13		
Total		118	168	62	36.9	0.7

^*a*^Middle Patuxent Environmental Area.

Average age of captured deer was 2.1 yr old ± 1.0. We captured 26 fawns (≤1 yr old), only 22% of captures. We collected 149 ticks from 29 individual deer across four of five trap sites. We collected 2 species (*Amblyomma americanum n* = 131 *and Ixodes scapularis n* = 18) of nymphs and adults (Table 3). We found 49% (*n* = 73, Left = 44, Right = 29) of ticks on the ears, 29.5% (*n* = 44) near the anus, and 21.5% (*n* = 32) on other parts of the body. We progressively collected more ticks each month ranging from 17 collected during February and 91 collected during April.

**Table 3.** Counts of species for ticks collected from live-captured deer

Tick species	*n*	% Nymph	% Male	% Female
*Ixodes scapularis*	18	11	33	56
*Amblyomma americanum*	131	37	37	26
Total	149	33.5	37	29.5

### Capture Success

Overall, 118 (94.4%) deer were captured with drop nets and 7 (5.6%) were captured in box traps. We did not have any recaptures with drop nets. However, on two occasions ear tags from previously trapped deer were found under the net most likely from deer that had escaped before they could be restrained. Only one recapture was recorded using box traps. We recorded 62 successful trapping events out of 168 total trapping events. Of our successful trapping events, we caught 1.9 deer per event. We recorded 78 trapping events in 2017 and 90 events in 2018. We recorded 62 successful trapping events with drop nets for overall success rate of 36.9% (Successful trap events/total trap events) or 1.4 trap nights per deer or .70 deer per trap night (Table 2). Trapping success rates per park ranged from 28 to 46% (Table 2). Pearson’s χ ^2^ test shows that trapping success was not significantly correlated to trap site based on the trapping effort at each park (χ ^2^ = 2.6086, df = 4, *P* = 0.6253).

**Fig. 3.**
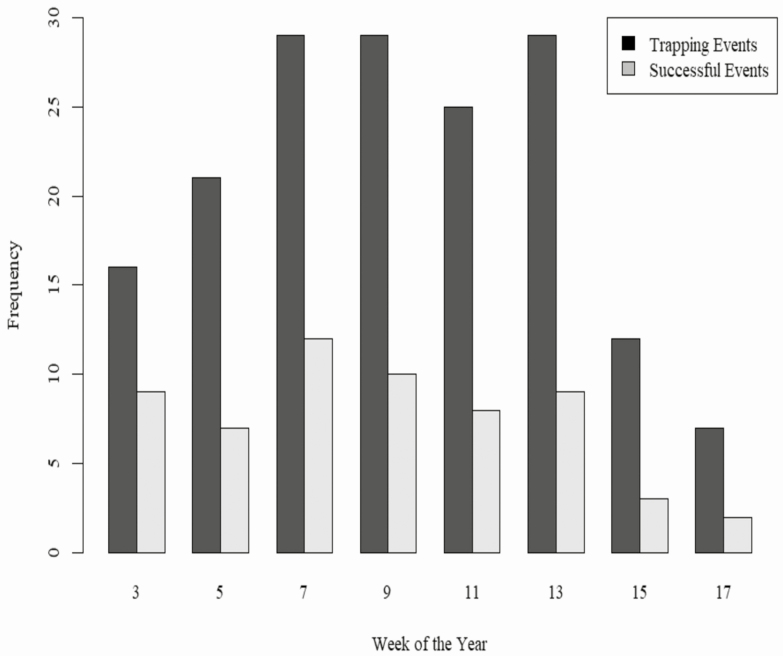
Histogram showing frequency of trapping effort per week from the beginning of the trapping season next to successful events by week across all parks and combined seasons.

**Fig. 4.**
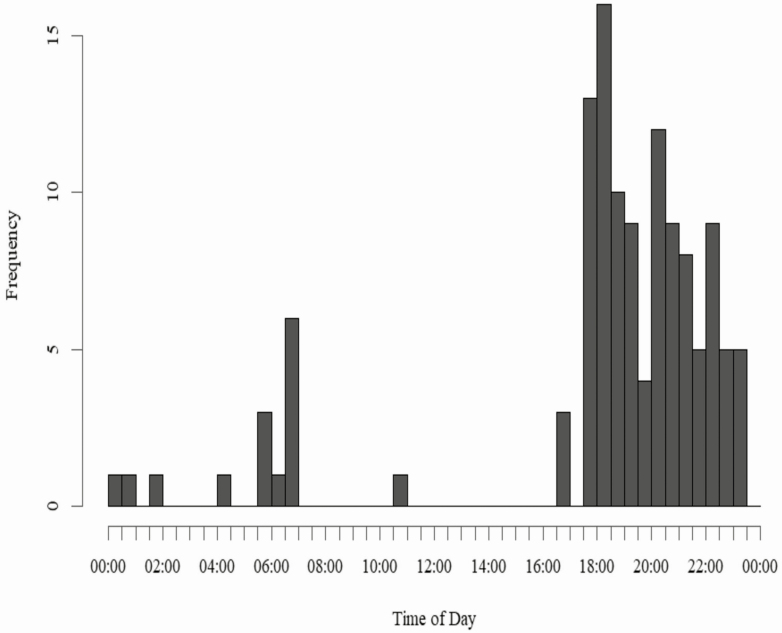
Histogram showing the frequency of times of successful captures across all trapping sites all trapping seasons.

Net trapping effort increased until peaking around February 20 through March 27 and then slowly declined (Fig. 3). The distribution of successful captures each week was not significantly different from the distribution of trapping effort (Two-sample Kolmogorov–Smirnov test: *D* = 0.12976, *P* = 0.4461). Time of capture primarily ranged from 1531 to 1938 hours and averaged 1800 hours (Fig. 4). On a few occasions, we captured deer before dawn which required trapping crews to arrive at midnight and trap until morning if camera traps showed peak deer activity from 0200 to 0500 hours. Average ambient temperature at capture was 7.6°C and ranged from −11.6 to 22.9°C.

### Spatial Analysis Results

Blandair and Cedar Lane were the most developed areas with 52.8 and 52.7% urban cover, respectively. For most of the trapping sites, <10% of the developed area was medium to high intensity or greater than 50% impervious surface. Cedar Lane was the only exception in which 15% of the developed area was medium/high intensity. MPEA and Wincopin Trails were the only trapping sites with majority forested cover (67.7 and 57.8%, respectively). MPEA and Wincopin Trails also had the smallest area of shrub/grassland cover (4.8 and 3.2%, respectively), whereas the other sites had 10–20% shrub/grassland cover. Cedar Lane was the only habitat analysis buffer with legitimate cultivated crops accounting for 12.4 ha (3.04%) of the land. Crops varied year to year during the study but were either soybeans, corn or hay/alfalfa pastures. Cedar Lane had the shortest Euclidean distances to several features, including major buildings and recreational fields, whereas MPEA had the longest average distances for these features (Table 4). The percent area of buildings within the buffer ranged from 3.8% at Wincopin Trails to 7.4% at Cedar Lane. Cedar Lane had the most area of recreational fields within the buffer (3.6%) as well. MPEA had the longest average Euclidean distance to roads among the trap sites (Table 4).

**Table 4.** Summary from GIS analysis of buffered areas around deer trapping sites

Trapping site	Cedar Lane	Rockburn Branch	Blandair Regional	Wincopin Trails/Savage	Middle Patuxent Environmental Area
Total buffer area (ha)	408.00	314.00	314.00	314.00	487.00
Capture success (%)	46.20	40.60	40.00	35.30	28.20
Urban cover (%)	52.70	45.10	52.80	37.90	27.40
Forest cover (%)	27.97	34.39	35.79	57.76	67.69
Grass cover (%)	19.31	20.38	11.18	3.20	4.81
Building cover (%)	7.39	5.19	5.67	3.77	3.90
Euclidean distance to buildings (m)	82.10	100.90	100.20	138.40	158.50
Recreational field cover (%)	3.62	0.99	0.94	1.21	1.31
Euclidean distance to recreational fields (m)	277.40	298.20	483.40	604.63	684.30
Road density	0.011	0.0073	0.014	0.0083	0.0060
Euclidean distance to roads (m)	67.56	60.62	76.03	92.60	159.79
Stream density	0.0023	0.0031	0.0023	0.0023	0.0041
Patch density	31.50	20.25	25.63	9.42	3.03
Landscape division index	0.48	0.48	0.49	0.43	0.03
Forest edge density	387.93	273.96	376.03	155.53	110.51

Total area reported as well as percent land cover classification. Distance, density or area of county features are also included for the study sites in Howard County, MD, 2020.

### Statistical Analysis

**Fig. 5.**
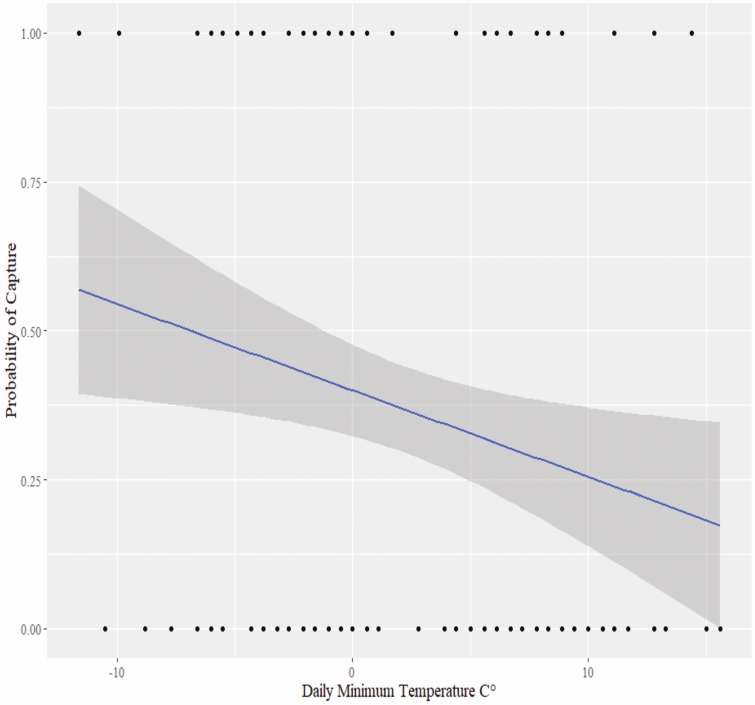
Regression of capture success versus daily minimum temperature (°C) (95% CI [−0.119, −0.013], RMSE = 1.13, *P* = 0.016).

Maximum value of *r* (0.39 for *daily snowfall* and *daily snow depth*) between any two independent weather variables indicated that correlation between these covariates would not affect GLM procedures by including all four variables (*daily minimum temperature, daily precipitation, daily snowfall, daily snow depth*) (Hiller et al. 2010). However, many spatial variables were highly correlated (*r* ≥ 0.7) limiting use in our regression models. After removing one variable of any pair that was highly correlated, the maximum value of *r* (0.62 for percent recreational field cover and road density) for any pair of spatial variables included in the model indicated that it would not affect GLM procedures. The input model included *weather covariates*, *Julian day*, *forest edge density, percent grassland/shrub cover, percent recreational field cover, and road density* within the buffers. Only *daily minimum temperature* was selected as a significant predictor of capture success (95% CI [−0.119, −0.013], RMSE = 1.13, *P* = 0.016, AIC = 225.41) (Table 5). Probability of capture increased with decreasing minimum daily temperatures (Fig. 5).

**Table 5.** Model selection results of the top five general linear models describing capture of white-tailed deer in suburban Maryland, 2017–2018

	Model	*K* ^*a*^	AIC	ΔAIC^*b*^	*w*_i_^*c*^
1	*Temp* ^*d*^	1	225.41	0.00	0.428
2	*SF* ^*e*^ *+Temp*	2	226.13	0.72	0.298
3	*SD* ^*f*^ *+SF+Temp*	3	226.85	1.44	0.208
4	*PRCP* ^*g*^ *+SD+SF+Temp*	4	229.50	4.09	0.055
5	*Sport* ^*h*^ *+PRCP+SD+SF+Temp*	5	233.18	7.77	0.009

^*a*^Number of model parameters.

^*b*^ΔAIC = relative difference to best performing model.

^*c*^AIC weight.

^*d*^Daily min. temp, °C.

^*e*^Daily snowfall, cm.

^*f*^Daily snow depth, cm.

^*g*^Daily precipitation, cm.

^*h*^% recreational field cover.

## Discussion

### Trapping Protocol

Generally, urban and suburban trapping operations require collaboration with state natural resource agencies to obtain any necessary permits. Likewise, local government agencies may request more involvement, depending on the location and ownership of the trapping sites. Trapping will most likely occur in high-use public places (parks, natural areas, open space), which may require additional permits, consideration of public use, and interference with other management activities. Because of the unique nature of suburban deer behavior and suburban habitats, modifications to typical rural deer trapping protocols may also be necessary (Peterson et al. 2003). In some cases, local or state managed white-tailed deer or other hunts may have priority over trapping events. These factors make proper, thoughtful trapping protocols in urban and suburban areas even more crucial.

### Public Perception and Engagement

The public’s close proximity to trapping sites and other research operations in suburban and urban studies may prompt more public engagement (Peterson et al. 2003, McCance and Baydack 2017). Researchers must anticipate the concerns and perceptions of residents in the area and be able to effectively convey project goals and operations to a diverse set of stakeholders. For the current project, we used multiple approaches for public engagement. Several attempts were made to inform the public about the goals of the project and anticipated management activities. The local natural resource agency organized several press releases to inform the public about the upcoming project and periodic updates throughout the project. Concurrently, crew members distributed project flyers and information pamphlets to neighboring homeowners near field sites. Conspicuous signage was placed at each trap to educate the public about the project and local codes prohibiting tampering with equipment. Because the overall project was related to controlling exposure to tick-borne diseases, local media coverage was also incorporated into the public outreach process. Nevertheless, face-to-face conversations were most successful in garnering interest and acceptance. As seen in other studies, our public contact was vital to the success of the project (Peterson et al. 2003).

While pedestrians would often walk by drop nets during operations, disturbance to trapping events from residents was not a major issue. In only one case did it significantly impact the trapping site. A drop net was vandalized during an inactive trapping period. Consequently, the net was not completely suspended, and a deer became tangled in the hanging net. Research staff were notified by residents and extracted the deer safely, but because of the lack of open space at this site suitable for trap set up and the observed vandalism, the site was removed from the study. Most issues encountered during trapping stemmed from equipment failure or user error.

### Site Selection

It is important to evaluate the environment in which trapping will take place, especially in suburban areas. Sites will present challenges and unique features that need to be incorporated into the study design. Parks and open space areas may have limited space to place traps like drop nets. There may be suitable grassy areas, but often these are associated with recreational fields, parking, or ongoing habitat management. Alternatively, maintained open space may be a right-of-way such as power, sewer, or gas lines. It is important to understand the restrictions and requirements for use of this space and to consult with the landowners or managers for access rights. Free-standing drop nets, which allowed more flexibility in trap placement without having to drive in support posts, should be considered in the initial study design (Peterson et al. 2003).

In general, all drop nets should be placed closer to the forest edge. Traps should be placed on level, dry ground free of debris, including rocks and roots. Trees and shrubbery may need to be removed if suitable open space does not exist. Access to trap locations for transporting trap equipment is necessary. However, at the very least the biologists should be able to easily transport necessary equipment to the trapping location on foot within a few minutes after capture. To complicate this issue, many of the suitable trap locations in suburban areas are public lands. Increased use of these lands by vehicular traffic may provoke public complaints. Special care should be used in these areas to reduce traffic in wet or muddy conditions and avoiding trail deterioration.

### Drop-Netting

Drop netting was the primary method to capture deer. We found drop netting to be a safe, quiet, and relatively efficient method for frequently capturing groups of deer (Conner et al. 1987, D’Eon et al. 2003, Peterson et al. 2003, Jedrzejewski and Kamler 2004).

Drop netting may be less biased towards younger deer. Fawns only comprised 22% of all drop net captures which is less than the range reported for other studies using clover traps reporting 40–66% of captures as fawns (Naugle et al. 1995, Haulton et al. 2001, Hiller et al. 2010, Cosgrove et al. 2012). We observed that smaller deer would enter under the net more readily only to be displaced by larger deer that dominate the bait pile. Having bait piles spread evenly enough to accompany multiple deer is imperative for catching groups at once. Many times, we dropped the net and deer caught just under the edge of the net would crawl out. A larger net would be beneficial to more effectively capture groups of deer, but we still recommend attempting to trap no more than five at a time (Conner et al. 1987, Pooler et al. 1997). At most, we caught four individuals during one trap event, which requires at least five personnel on site to maintain safe handling and prevent increases in the likelihood of capture-related mortalities (Conner et al. 1987).

Disadvantages of drop nets include limited ability to select specific deer by sex, age, or other parameters. Drop nets are also conspicuous and must be left in the environment for deer to become acclimated, making them vulnerable to vandalism and weather-induced wear. Although drop nets are generally considered safe, netting poses a risk to antlered bucks that may get caught in the netting and can cause premature antler removal. Nets can also damage new antler growth if trapping is conducted into the spring or summer. Nets may also interfere with immediate positioning of deer in sternal recumbency. Immobilized deer need to be untangled and removed from the net in a timely manner. Proper drop net set up and maintenance is critical to success.

We captured most deer at dusk. Prime capture time seemed to occur later at heavily forested sites, but we still recommend setting traps before dusk. Our trapping protocol required the use of night vision or FLIR units to detect deer under nets. In the current study, daily use of parks by residents became more frequent towards the spring months, but throughout the project pedestrian or bike traffic was common in the parks from dawn until dusk. Sports activity was also a factor, and recreational field lights remained on into the night. The continuous presence of people in and around trapping areas prevented trapping from occurring until after the parks were closed, even though camera traps showed that deer occasionally visited box traps and drop nets during daylight hours. In less populated parks, it is recommended that traps are prepared, and operators hidden at least an hour before dusk. Deer at more developed parks seemed to exhibit less avoidance behavior to human activity. So, in heavily used parks, 15 min to a half an hour may be enough due to deer habitation to human activity. Some nets were erected right next to walking trails, and late-night pedestrians would scare deer from approaching the net. However, deer at more urban parks would often return within 15–30 min after the pedestrian left the area. Deer at the more secluded, forested parks seemed weary towards human activity and would not return after being startled. Hunting blinds can help reduce motion of technicians, but we recommend setting blinds into the forest edge and hiding it well because often the blind would draw attention from approaching deer.

For trapping in rural areas, pre-baiting for a period of weeks is often recommended. It was our experience that in some areas deer came to bait the day after traps were erected. Deer should be given several days to acclimate to nets, and to learn that bait is routinely available, but long acclimation periods may not be necessary with suburban deer. White-tailed deer exhibited a degree of avoidance behavior to bait with other wildlife under the net. These interactions may have had an influence on trapping success. Birds, squirrels, raccoons, foxes, and rabbits were documented visiting trap sites to access bait. It was observed that attendance by foxes or raccoons at bait sites under drop nets would inhibit deer in the area from foraging under the net.

### Box Trapping

Netted cage traps have the advantage of being lightweight, portable, fairly inconspicuous, and the only passive trapping option that can be placed in smaller locations. These traps can be set at specific times of the day, and placed in more wooded areas, not requiring open space. However, these traps do tend to capture younger deer (Hiller et al. 2010), and male captures may occur less frequently than female (Hiller et al. 2010). However, a majority of our box trap captures were male. In our study, four box traps were used to supplement our primary trapping effort. Box trapping greatly increased our trapping effort and minimally increased capture success (seven captures). Most of the effort in box trapping was attributed to traveling from the field station to trapping sites and not from checking individual traps, which were often placed within 100 m of one another. In the future, we would either not use box traps or double the number of traps deployed to increase chances of capture without much effect on trapping effort (Jordan et al. 1993). Furthermore, we recommend using alternative bait to corn and apples in box traps as these received heavy non-target animal disturbance. Other trapping programs have used alternative baits such as hay/feed mixtures (Barrett et al. 2008).

### Tick Collection Protocol

The distribution of ticks on their deer hosts is often congregated towards forelimbs, neck, and head, allowing rapid assessment on tick abundance and reliable sampling zones for surveillance efforts (Kiffner et al. 2010). Individual deer in this study would be examined for ticks by one or more technicians, but no formalized search effort was recorded. We primarily searched for and removed ticks on the ears, head, and anus but did a full body assessment and removed ticks from the axilla and abdomen region. However, maintaining anesthetized deer in sternal recumbency was a priority to ensure deer safety during processing, which restricted search time on the underside of deer.

Adult ticks were found on ears, anus, and other parts of the body. Interestingly, nymphs were never found in the anus region. We recommend standard inclusion of the anal region in search efforts as it is easily accessible and lacks hair that might conceal attached ticks.

Tick distributions within local environments can be highly patchy, highlighting the need to sample at multiple locations for a better understanding of prevalence or abundance within communities (Pardanani and Mather 2004). In this study, low tick numbers were expected because trapping occurred during winter months with cooler temperatures to mitigate hyperthermia in deer. Nearly 90% (*n* = 134) of removed ticks came from 15 deer from one park. Preliminary data showed that the park in which most ticks were collected from deer in this study may have generally higher tick densities along with low mouse numbers, indicating a potential lack of host diversity. Meanwhile, the larger USDA-ARS project conducted tick dragging and small mammal trapping for additional collection of ticks during spring, summer, and fall.

Best results for tick collection occur on live or freshly deceased hosts since some parasites detach from expired hosts, which may bias samples removed from roadkill and hunter-harvested hosts (Tsunoda 2014). Trapping during peak tick activity season may increase the probability of collecting ticks on captured deer. We collected more ticks as the season progressed even though successful captures and trapping effort waned towards the end of the season. Unfortunately, higher ambient temperatures decrease capture probabilities and significantly increase capture myopathy and capture-related mortalities.

### Capture Success

We cannot say one project was more successful than the other since many factors influence trap success both locally and regionally (Garrott and White 1982, Barrett et al. 2008, Hiller et al. 2010, Cosgrove et al. 2012). Furthermore, comparisons of capture success should be considered loosely between different studies as some have used different trapping methods, different definitions of ‘trap nights’, different definitions of ‘trap success’, and often have incomplete data recorded on trapping effort for some seasons. Most studies report capture success as number of deer captured per trap night or number of trap nights per deer, but these studies heavily relied on box trapping which is not designed to capture multiple individuals at the same time like drop netting (Garrot and White 1982, Morgan and Dusek 1992, Jordan et al. 1993, Naugle et al. 1995, Barrett et al. 2008, Hiller et al. 2010, Cosgrove et al. 2012). If we use the number of deer per trap-night, our overall success rate is nearly 1.0 for some individual sites. However, a majority of trap nights we failed to capture deer. False triggers, released captures, non-target animal disturbance on traps was not accounted for in these estimates. Several trapping events were interrupted by electrical failures from incorrectly wiring the drop net or broken wires from fraying. Cold weather also drained power from the electronic equipment quicker than usual.

Since drop netting often catches multiple deer at a time, we felt it was more accurate to calculate capture success as the ([number of successful trap events]/[total number of trap events]). With this statistic, our success rate for drop netting was 0.37 or at least one deer on 37 of 100 trap nights. When calculated as deer/trap night, our capture rate is 0.70. We were successful 36.9% of the time and captured 1.9 deer per successful event. In a similarly designed, yet rural study, Conner et al. (1987) reported 48.7% (55 drops/113 trapping attempts) success rates using drop nets and caught 3.2 deer per drop. However, Conner et al. (1987) used larger drop nets in agricultural areas with reported deer densities of 36/km^2^.

We were restricted to trapping on county owned land but other studies have had success on private residences (Jordan et al. 1993, Peterson et al. 2003). These secluded properties, especially on larger lots (>1 ha), are prime refugia for urban deer. Including corporate lands and holdings, non-hunted state and county parks, nature preserves and easements, and municipal open space would be other potential trapping sites in suburban or urban areas (Ellingwood and Kilpatrick 1995).

### Spatial Analysis

Past papers quantifying and evaluating trap success typically have large study areas or generally describe the trapping locations. This is not as useful to urban managers where land use can change drastically in short distances. Our aim was to provide a successful urban/suburban trapping protocol, along with a small scale ≤1 km distance evaluation of habitat for urban trapping programs. The least successful trapping sites were MPEA (28.2% capture success, 0.38 deer/trap-night) and Wincopin Trails (35.3% capture success, 0.57 deer/trap-night) which were also the most forested (67.7 and 57.8% respectively) and had the least amount of forest edge habitat (Table 3). The most successful park was Cedar Lane (46.2% capture success, 0.92 deer per trap-night), and Rockburn and Blandair both had similar capture success 40% of trap events (0.94 deer per trap-night, 1.0 deer per trap-night, respectively) (Table 2). Rockburn, Blandair, and Cedar Lane trapping sites had the most available shrub/grassland habitat. Cedar Lane and Blandair were the most urban with 52.7 and 52.8% urban cover respectively but also had the highest densities of forest/open edge habitat.

Even though contiguous forest is limited in Howard County, Maryland, and forest patches were small and interspersed, trapping success still had an inverse relationship with the area of forest cover. The suburban areas in our study with the best capture success exhibited higher amounts of forest edge habitat and not necessarily contiguous forest habitat. They had smaller habitat patches, denser building cover, and shorter distances to urban features such as buildings, roads, and recreational fields. This is certainly something managers and researchers should keep in mind when selecting trapping sites. Furthermore, because white-tail deer home range sizes decrease in size when there is more forest edge habitat, managers will likely have higher deer densities in these highly fragmented Parks (Walter et al. 2018). Those higher densities, coupled with human habituation, may have led to the higher capture success in this study.

### Weather

Poor weather (i.e., below freezing, snow) has been linked to decreased activity in white-tailed deer. This is an energy conservation strategy when natural forage is low and may not be as advantageous when artificial food sources are readily available because of trapping (Verme 1973, Moen 1976, Taillon et al. 2006). Deer may increase activity and movement towards a bait pile or artificial food source during similar conditions since it is easily accessible food (Taillon et al. 2006). We documented increases in probability of capture as daily minimum temperatures decreased. Hiller et al. (2010) found similar effects of minimum temperatures on capture success in more northern latitudes; however, we did not detect any significant effect of snowfall or snow depth on capture success in our model. However, Maryland has less severe and infrequent winter storms and these covariates may be less reliable in this region for predicting capture success. Other weather covariates not accounted for in our analysis, such as wind velocity or barometric pressure, may influence capture success as well.

### Trapping Considerations Summary

If live trapping white-tailed deer is necessary to reach management or research objectives in urban or suburban areas, we recommend the following:

- Develop significant public outreach before fieldwork occurs
- Connectivity between parks and edge density habitat patches will greatly influence deer distribution and deer behavior throughout the area.
- Develop an urban/suburban specific trapping protocol, with concentrated drop net trapping and preparations for significant pedestrian/human interactions
- Small, human-developed parks are often the most productive trapping sites
- Cold weather and snow likely drives trapping success, followed by presence of recreational fields
- When collecting vectors, such as ticks, as appropriate, do full-body searches.

## Supplementary Material

ieaa044_suppl_Supplementary_Material

## Notes

Disclaimer: This article reports the results of research only. Mention of a proprietary product does not constitute an endorsement or a recommendation by the USDA for its use. The USDA is an equal opportunity provider and employer.
