# Supplementary material for: Trapping White-Tailed Deer (Artiodactyla: Cervidae) in Suburbia for Study of Tick–Host Interaction
Source: J Insect Sci. 2020 Nov 2;20(6):8. doi: 10.1093/jisesa/ieaa044 (PMC7604841; doi:10.1093/jisesa/ieaa044)
Supplement: ieaa044_suppl_Supplementary_Material [file ieaa044_suppl_supplementary_material.docx]

**Supplementary Material**

Table 6. List of supplies used in the deer trapping protocol. Includes examples and grouping.

| Item | Purpose | Example | Grouping^1^ |
| --- | --- | --- | --- |
| Emergency and Medical History Information | For emergency purposes: Relevant information for each crew member as well as information on immobilization and reversal agents used. | Formulation of immobilizing drugs, emergency numbers, hospital locations | All |
| Extra Batteries | Extra set of batteries for all devices in the field | Varies | All |
| Headlamp | Visibility at night | Varies | All |
| Ratchets, wrenches, screw drivers | Assemble/disassemble and repair field equipment | Varies | All |
| Signage | Inform public on management activities and local regulations | Metal signs near traps, flyers, press releases, media coverage | All |
| Air-activated heat packets | Keep cold sensitive items warm while in the field (i.e. immobilizing drugs) | HotHands® Hand Warmers (Home Depot SKU 513219) | Capture |
| Batting Helmet | For use by personnel in clover trapping during restraint | Rawlings® Softball Helmet w/ mask | Capture |
| Box-trap | Live-capture | Medium Wildlife Capture Services Box-trap | Capture |
| Car Battery Charger | Charge 12 volt batteries | Varies | Capture |
| Drop-Net | Live-capture groups of animals | 40' x 40' Wildlife Capture Services Drop-Net System | Capture |
| Drop-Net Batteries | Power electromagnets on drop-net | Deep Cycle marine 12 volt battery | Capture |
| Heavy Duty T-Posts | Secure traps and trap support posts to ground | 6ft. Green Steel Fence T-Post (Home Depot SKU 373311) | Capture |
| Hunting Blind | Hide net operators, reduce motion | Ameristep Doghouse Blind (Bass Pro Shops SKU 2581256) | Capture |
| Loppers/Machete | Clear vegetation from trap locations | Fiskars® Bypass Lopper (Home Depot SKU 643278) | Capture |
| Metal Chain | Attach traps and support posts to T-posts | #2/0 Stainless Steel Straight Link Chain (Home Depot SKU 263436) | Capture |
| Thermal Imaging Devices | Visibilityfor trapping at night | FLIR Systems Scout II 240 | Capture |
| T-post driver | Drive T-posts into the ground | Metal Fence Post Driver (Home Depot SKU 108235) | Capture |
| T-post remover | Easily remove T-posts from ground | Post Pull'R (Home Depot SKU 517895) | Capture |
| Two-Way Radio | Allows direct communication between crew members; alert crew of captures | Midland® GXT1000VP4 Two-Way Radio | Capture |
| Whole Kernel Corn/Apples | Bait | Varies | Capture |
| Camera Traps | Monitor trap sites, activity at bait sites | Moultire® M-888 Mini Game Camera | Monitoring |
| Cellular Camera Traps | Monitor trap sites and activity at bait sites; remotely sends pictures | SPYPOINT® Link 3G | Monitoring |
| Lock Boxes | Metal protection housing for camera traps to deter damage or theft | SPYPOINT® SB-Pro Steel Security Box | Monitoring |
| Python Locks | Secure camera traps and other field supplies | Python Adjustable Lock (Home Depot SKU 577100) | Monitoring |
| Antibiotic Cream | Treat any wounds sustained during capture | Neosporin® | Processing |
| Biohazard Bags | Dispose of biological waste | Varies | Processing |
| Captive Bolt-Gun | Euthanize animals | BLITZ® Captive Bolt Gun Kit (QC Supply # 140760) | Processing |
| Digital Thermometer | Monitor rectal temperature | Varies | Processing |
| Ear Tag Applicator | Applies ear tag to animal | Destron Fearing™ Duflex® ProGrip™ II Universal Applicator (QC Supply Part # 140330) | Processing |
| Ear Tags w/ backing | Uniquely identify captured animals | Destron Fearing™ Duflex® Medium ID Ear Tags (Valley Vet Supply Item 20713) | Processing |
| Emergency Blankets | Warm up hypothermic animals | Emergency Blanket 87" x 59" (MCR Medical SB-1001-001) | Processing |
| EPI Pen Containers | Safe storage for syringes containing drugs while in the field | EPI PEN® Jr. plastic holster case | Processing |
| Ethanol Vials | Vials filled with 70% ethanol for collecting ticks | 15mL tubes w/ 5mL of ethanol | Processing |
| Face Blinds | Reduces stress to captured animals | Full face hood/mask (Wildlife Capture Equipment SKU HD002-M) | Processing |
| Fishing Tackle Box | Organize, transport, and store processing equipment | Plano® XL 3-Tray Box (Bass Pro Shops SKU 1719875) | Processing |
| Forceps | Remove attached parasites | Varies | Processing |
| Gauze Pads | Clean, dress wounds during processing | Varies | Processing |
| Ice | Cool down hyperthermic animals | Varies | Processing |
| Immobilizing Agent | Used to safely process animals for extended periods of time | BAM™ Kit Wildlife Pharmaceuticals | Processing |
| Insulated Container | Preserve temperature sensitive items (i.e. drugs, samples) | Varies | Processing |
| Isopropyl Alcohol | Used to cool down hyperthermic animals; sterilize equipment | Varies | Processing |
| Measuring tape | Record morphometric data | Varies | Processing |
| Narcan® | Reverse effects of opioids in instance of accidental human exposure | Narcan®(naloxone HCL) Nasal Spray (ADAPT Pharma, Inc) | Processing |
| Nitrile Gloves | Processing and handling of all wildlife animals | Varies | Processing |
| Oxygen Tanks | Provide supplemental oxygen to anesthetized animals | Size E Cylinder with Regulator (AirGas #OX USPEAWB) | Processing |
| Permanent Markers | Marking vials, tags, and data sheets | Varies | Processing |
| Pulse Oximeter | Monitor blood oxygen saturation | SurgiVet® v1030 (Smiths Medical) | Processing |
| Reversal Agent | Reverse animals from anesthetization | Atipamezole and Naltrexone Wildlife Pharmaceuticals | Processing |
| Saline Solution | Flush out debris from any wounds | Varies | Processing |
| Saw | Clear vegetation or remove antlers in emergency situation | Hacksaw (Home Depot SKU 1000032953) | Processing |
| Sharps Container | Dispose of used syringes and needles | Varies | Processing |
| Split Cannula | Administer oxygen to multiple animals at once with one tank | Varies | Processing |
| Syringes w/ needles | Deliver immobilizing drugs | 5mL Nipro Luer Lock Syringes 21 gauge 1 inch needles | Processing |
| Tarps | Thermal insulation for processing animals | Varies | Processing |
| Water Coolers | Store and transport ice | Varies | Processing |
| Wax Pens | Mark injection site to avoid contact after delivering drugs | Raidex Prima Tech marking sticks (QC Supply #140150) | Processing |

Disclaimer: The following is a description of equipment used in this suburban white-tailed deer trapping study, but is not meant to be comprehensive or absolute. Methodology for processing and handling deer may differ based on experience, IACUC protocols, study design and objectives.

^1^Supplies are grouped into four main aspects of trapping: *Capture*-preparation and construction of traps; *Monitoring*-monitoring of trap sites and animal activity; *Processing*- safely processing and collecting samples from immobilized animals; *All*- imperative for all aspects of trapping
